# Post-Chemotherapy Retroperitoneal Lymph Node Dissection for Metastatic Testicular Cancer at a National Referral Centre

**DOI:** 10.3390/cancers17040608

**Published:** 2025-02-11

**Authors:** Konstantinos Evmorfopoulos, Panagiotis J. Vlachostergios, Georgios Chasiotis, Anastasios Karatzas, Ioannis Zachos, George Koukoulis, Konstantinos Dimitropoulos, Louis L. Pisters, Vassilios Tzortzis

**Affiliations:** 1 Department of Urology, Faculty of Medicine, School of Health Sciences, University of Thessaly, University Hospital of Larissa, 41100 Larissa, Greece; 2 Department of Medical Oncology, IASO Thessalias Hospital, 41005 Larissa, Greece; 3 Department of Medicine, Division of Hematology and Medical Oncology, Weill Cornell Medicine, New York, NY 10065, USA; 4 Department of Pathology, Faculty of Medicine, School of Health Sciences, University of Thessaly, 41100 Larissa, Greece; 5 Department of Urology, Aberdeen Royal Infirmary, Aberdeen AB25 2ZN, UK; 6 Department of Urology, Division of Surgery, The University of Texas MD Anderson Cancer Center, Houston, TX 77030, USA

**Keywords:** adjunctive procedures, chemotherapy, complications, metastasis, pc-RPLND, retroperitoneum, testicular cancer

## Abstract

Objective: To assess the safety of open PC-RPLND at a high-volume national referral centre over the course of several years. Materials and Methods: A retrospective chart review of patients with testicular germ cell tumours (TGCTs) who underwent PC-RPLND at our institution between 2008 and 2023 was conducted. Patient demographics, clinical characteristics, intraoperative and postoperative parameters and adjunctive procedures were recorded. ClassIntra and Clavien Dindo classifications were used to assess intraoperative and postoperative complications, respectively. Results: In total, 165 patients were studied. The median (Q1–Q3) age of patients was 30.5 years (24.75–38.25), and the median maximum diameter of retroperitoneal masses was 50 mm (26.75–81.25). The most common adjunctive procedure was synchronous nephrectomy (*n* = 18, 11%) followed by vascular procedures (n = 7, 4.3%), ureteric reconstruction (n = 7, 4.3%), and partial hepatectomy (n = 3, 1.9%). Intraoperatively, 20, 8 and 1 patient had a grade I, II or V complication, respectively, according to the ClassIntra classification. The median estimated blood loss was 300 mL (120–740), the median duration of the procedure was 4.9 h (4–6 h) and the median length of stay was 8 days (7–10 days). Histopathological examination of the resected specimen showed teratoma in 51.9% of patients, followed by fibrosis/necrosis in 39.5%. A total of 40 patients (24.7%) experienced at least one complication. Conclusions: PC-RPLND is a complex operation, often accompanied by adjunctive surgical procedures and therefore must be conducted in high-volume referral centres to ensure safety and minimise complications.

## Simple Summary

Testicular cancer often requires additional treatment after chemotherapy to remove any remaining tumour tissue, particularly in the abdominal region. This procedure, called post-chemotherapy retroperitoneal lymph node dissection (PC-RPLND), is complex and needs to be performed by skilled teams in specialized centres. This research examined the outcomes of this surgery in a high-volume national referral centre over several years. By studying over 160 patients, it was found that while the surgery is challenging and sometimes requires additional procedures like kidney removal, it is safe when conducted by experienced teams. These findings emphasize the importance of referring patients to specialized centres to ensure optimal care and minimise complications.

## 1. Introduction

Testicular cancer is the most common cancer among young men between 15 and 40 years of age. The vast majority (98%) of all testicular cancers are germ cell tumours (TGCT) [1,2,3,4].

Current multimodal treatment, when necessary, provides a 10-year cancer-specific survival rate of >95% [5]. Survival highly depends on the timely and thorough quality of delivery of surgery and chemotherapy. Despite the effectiveness of platinum-based chemotherapy, 25% of patients have a residual retroperitoneal mass post-completion of systemic treatment [6]. The objective of retroperitoneal lymph node dissection (RPLND) is to remove persistent lymph nodes as they contain teratoma or viable cancer in 30–40% and 10–20% of patients, respectively [7]. Teratomas require special attention as they are chemotherapy-resistant, may relapse years after initial treatment [8], and have an inherent risk of transforming into a somatic-type malignancy, which is often incurable [9]. Complete resection after initial chemotherapy can increase long-term disease-free survival up to 95%. The overall long-term relapse rate in patients undergoing RPLND ranges between 6% and 9% [10].

Due to the complex anatomy of the retroperitoneal space and the extensive desmoplastic reaction often seen after chemotherapy, especially in seminoma, pc-RPLND is a challenging surgical procedure that should be performed by experienced surgeons familiar with the retroperitoneal anatomy and able to perform complex surgical procedures on the vascular and intestinal structures [11,12]. In addition, pc-RPLND has been associated with higher morbidity compared to primary RPLND, with complication rates reported between 1.2 and 23.3% [13,14]. The associated morbidity is mainly attributed to the adjunctive procedures that may be required intraoperatively, such as resection of the kidney, vena cava, aorta, or bowel. Approximately one-third of patients will require an additional procedure that can lead to a prolonged hospital stay [15]. An attempt to minimise complications requires through knowledge of the retroperitoneal space, the relevant anatomical landmarks and resection templates, as well as multidisciplinary input intra- and postoperatively based on commonly agreed protocols.

This retrospective study evaluated the safety of pc-RPLND for metastatic testicular cancer at a national referral centre. It also provides a detailed description of its clinical effectiveness according to histological subtypes, disease stage, sites of metastatic disease, and response to chemotherapy.

## 2. Materials and Methods

### 2.1. Study Population

A retrospective chart review protocol was approved by the Institutional Review Board (Protocol No: 22978/16-05-2023). The departmental testicular cancer database was retrospectively reviewed, and all consecutive patients with testicular cancer who underwent pc-RPLND from 2008 to 2023 were included in the analysis. The indication for surgery was any residual mass ≥1 cm with normal or plateaued tumour markers after the completion of chemotherapy. RPLND was performed via an open transperitoneal approach by two senior uro-oncology surgeons. Boundaries of surgical dissection were the renal vessels cephalad, the ureters laterally, and the bifurcation of the iliac arteries caudal (full bilateral template). A modified unilateral template was used on selected patients based on the size of the retroperitoneal mass, its location in the retroperitoneum, its anatomical relation to other structures and the laterality of the primary testicular tumour. Right-sided modified template resection encompassed the precaval, paracaval, retrocaval, and interaortocaval regions. It also included the area lateral to the common iliac vessels, with the ureter crossing the iliac vessels serving as the caudal boundary and the ureter itself acting as the lateral boundary of dissection. The cranial boundary was defined by the renal vein, except in cases where retrocrural or suprahilar lymph node involvement was present. Left-sided modified template resection involved the preaortic and para-aortic regions up to the inferior mesenteric artery, as well as the retroaortic area. The crossing of the ureter over the iliac artery marked the caudal boundary, while the ureter served as the lateral boundary of dissection [11].

In all cases, a complete medical assessment was performed, including measurement of serum tumour markers (alpha-fetoprotein, AFP; β human chorionic gonadotropin, βHCG; and lactate dehydrogenase, LDH). Moreover, all patients were offered restaging chest and abdominal/pelvic computed tomography (CT). The maximum transverse diameter of the retroperitoneal mass on CT before and after chemotherapy completion was recorded.

All patients had received systemic chemotherapy before undergoing RPLND. The most frequently used chemotherapeutic regimens were bleomycin, etoposide, and cisplatin (BEP, 3–4 cycles) or etoposide and cisplatin (EP, 4 cycles), while some patients received 2–4 cycles of paclitaxel, ifosfamide, and cisplatin (TIP), cisplatin etoposide ifosfamide (VIP) or combinations of these chemotherapy regimens.

The study recorded any adjunctive procedures performed during surgery. Intraoperative adverse events (iAE) and postoperative complications were systematically recorded and classified using the ClassIntra v1.0 and the Clavien-Dindo classification systems [16,17]. Complications were recorded either during regular follow-up clinic visits or via telephone follow-up appointments. The follow-up protocol included complete blood count, blood biochemistry, including AFP, βHCG, and LDH levels every 3 months, and chest–abdomen–pelvis CT scans every 6 months for a 2-year period and ten once a year in line with the relevant European Urology Association guidelines [18].

### 2.2. Statistical Analysis

Patient demographics, clinicopathological characteristics and intra- and postoperative complications were analysed with the Statistical Package for Social Sciences (SPSS) v.29 program. Continuous variables with normal distribution are presented as means ± standard deviation (SD) and non-normally distributed data as medians (Q1–Q3), while categorical variables are presented as proportions.

The statistical analysis involved the use of the chi-square test to assess associations between categorical variables. Chi-square tests were employed to examine relationships among the type of RPLND procedure and postoperative complications, allowing for the determination of statistical significance.

## 3. Results

### 3.1. Preoperative Evaluation

A total of 165 consecutive pc-RPLNDs were performed at the University Hospital of Larissa, Greece during the study period. All patients were referred to our centre after completion of platinum-based chemotherapy and, in the majority of the cases, their pre-operative serum biomarkers were within the normal range. Seven patients (4.2%) had elevated tumour markers (plateaued), while another 4 (2.5%) underwent salvage RPLND after the completion of salvage chemotherapy. An assessment of disease stage showed that 16 (9.7%), 31 (18.8%), 49 (29.7%) and 69 (41.8%) patients were diagnosed with stage IIA, IIB, IIC and III disease, respectively. The median (Q1–Q3) maximum diameter of retroperitoneal masses on CT scans prior to RPLND was 50 mm (26.75–81.25 mm). Patients’ demographics and baseline clinical characteristics are presented in Table 1.

### 3.2. Intraoperative Parameters and Adjunctive Procedures

Three patients were excluded from final analysis; two due to unresectable mass identified intraoperatively and one due to intraoperative death secondary to massive hemothorax. Of the remaining 162 patients, 125 (77%) were offered full bilateral dissection, while the remaining 37 patients (23%) had template dissection.

The median (Q1–Q3) duration of the procedure was 4.9 h (4–6 h), and the estimated blood loss was 300 mL (120–740). According to the ClassIntra v1.0 classification of intraoperative adverse events (iAE), 20 patients (12.4%) had a grade I intraoperative complication (mostly due to bleeding above average) with no need for additional intervention, eight patients (5%) had a grade II complication (ureteric injury in seven patients with ureteric stent placement, and major vessel injury in one patient who needed stenting of the left internal iliac artery), while, as mentioned above, one patient died intraoperatively due to massive hemothorax and was classified as a grade V complication.

In 38/162 patients (23.4%, at least one additional procedure was required. More specifically, 18/162 (11.1%) patients underwent synchronous nephrectomy, one patient (0.6%) underwent renal autotransplant, three patients (1.9%) required intraoperative insertion of ureteric stent, while ureteric reconstruction in the form of transureteroureterostomy (TUU) or ureteric replacement with ileal graft were performed in three (1.9%) and one patient (0.6%), respectively. Vascular stenting of the left internal iliac artery was required in one case (0.6%), and excision of IVC was performed in five cases (3.1%) due to thrombosis. One patient (0.6%) was offered a total reconstruction of IVC and aorta from the level of the renal vessels to common iliac vessels with the use of synthetic grafts, to allow for complete resection of residual mass. Additionally, three patients (1.9%) with visceral metastatic disease underwent partial hepatectomy, one (0.6%) underwent hepatic metastasectomy with radiofrequency ablation, while partial vertebrectomy was offered to one (0.6%) additional patient. All intraoperative parameters are shown in Table 2.

### 3.3. Postoperative Parameters and Complications

Within the first 30 postoperative days, 40 out of 162 patients (24.7%) experienced at least one complication. According to the Clavien-Dindo Classification of postoperative complications, the most common Grade I complication was excessive lymphatic leakage in 28/162 patients (17.3%), requiring prolonged percutaneous drainage post-discharge. Moreover, three (1.9%) patients developed fever, managed with conventional antipyretics. Grade II complications were reported in a total of eight (4.9%) patients: four patients had an infection (chest or wound infection) requiring antibiotics, ileus was recorded in two cases and two additional patients suffered episodes of pulmonary embolism/deep vein thrombosis. One patient developed wound dehiscence and was managed with surgical intervention under general anaesthesia (Grade IIIb). No grade IV or V complications were reported.

Complications were three times more common in patients with full bilateral resection templates compared to those who were offered template resections. (chi-square: 4.969, df: 1, *p* = 0.026, OR = 3.34 with 96% CI: 1.102–10.101) (Table 3). The median (Q1–Q3) length of hospital stay was 8 (7–10) days.

Histopathological examination of the pc-RPLND specimen showed that teratomas were the most frequent finding (83/162, 51.2%), followed by fibrosis/necrosis without residual viable tumour (64/162, 39.5%). The retroperitoneal lymph nodes of two patients contained rhabdomyosarcoma, nephroblastoma and adenocarcinoma components due to the progression of immature teratoma. Detailed histopathological findings of the entire pc-RPLND cohort and a summary of postoperative parameters are shown in Table 4.

### 3.4. Clinical Findings and Surgical Outcomes in Seminoma Patients Undergoing PC-RPLND

Out of the total cohort, 11 out of 165 individuals were identified with seminoma upon initial histopathological evaluation. The median age at diagnosis was 38 years (28.75–45.5 years). Among these patients, six (54.5%) underwent BEP chemotherapy, three (27.3%) received EP, and two (18.2%) were treated with a combination of BEP and TIP. Staging assessments revealed that two patients (18.2%) had stage IIA, another two (18.2%) had stage IIB, four (36.4%) presented with stage IIC, and three (27.3%) were diagnosed with stage III disease. The median (Q1–Q3) maximum diameter of retroperitoneal masses observed on pre-RPLND CT scans was 63.5 mm (33.75–107 mm).

The majority of patients (9/11; 81.8%) underwent complete bilateral pc-RPLND, while two individuals (18.2%) underwent a modified template procedure. The median (Q1–Q3) duration of the procedure was 5.5 h (4.9–7.0 h). According to the ClassIntra v1.0 classification system for intraoperative adverse events (iAEs), seven patients (63.6%) experienced grade I complications, primarily due to above-average bleeding, which did not necessitate additional interventions. Grade II complications occurred in two patients (9.1%) and were attributed to ureteric injuries that required stent placement.

Additional surgical procedures were performed in four out of 11 patients (36.4%). Specifically, synchronous nephrectomy was conducted in two cases (18.2%), intraoperative ureteric stent placement was required in one case (9.1%), and partial vertebrectomy was carried out in another patient (9.1%). Within the first 30 days postoperatively, four patients (36.4%) encountered at least one complication. Based on the Clavien-Dindo classification, two patients (18.2%) experienced excessive lymphatic leakage (classified as Grade I), and two others (18.2%) developed wound infections (classified as Grade II). The median (Q1–Q3) length of hospitalization was 10.5 days (7.25–12.75 days). Histopathological analysis of the pc-RPLND specimens revealed that fibrosis or necrosis was the predominant finding, occurring in eight patients (72.7%), while residual seminoma was detected in three patients (27.3%).

## 4. Discussion

Centralisation of care for patients with metastatic testicular cancer has been associated with better outcomes in terms of postoperative complications, disease-free survival and overall survival [19]. The current study assessed the safety outcomes of post-chemotherapy RPLND for the management of testicular cancer over a 15-year period at a national referral centre.

The present study shows that the most common additional procedure in the patient cohort was total nephrectomy performed in one-tenth of patients, followed by vascular and ureteric reconstructions. The need for additional procedures is higher in bigger masses (particularly >5 cm in size) as well as in intermediate- and poor-prognosis IGCCCG patients [20]. In this study, this correlation was not examined since the majority of the patients in this cohort had retroperitoneal masses with a median maximum diameter of 5 cm. The rate of additional interventions in this study was 23.5%, confirming the complexity of PC-RPLND as a result of extensive tissue plane distortion of by the enlarged mass, and direct infiltration of the tumour to local structures, such as the ureter, aorta, and vena cava. In general, the frequency of additional procedures ranges between 13% and 38%, with nephrectomy and vascular procedures representing the most common ones [15,21,22,23,24]. In the presence of tumour extension into the renal hilum, nephrectomy is inevitable, and histopathological examination of the resected kidney shows pathological infiltration in more than half of patients. Malignant infiltration of the lumbar spine is very rare (3–9% of GCT patients) and only a few vertebrectomy cases have been reported [25,26,27]. In the current study, one patient underwent partial lumbar vertebrectomy secondary to tumour infiltration.

Additionally, four cases of partial hepatectomy and radiofrequency ablation of liver metastases are reported. Overall, reports of liver metastasectomy in TGCTs remain scarce. According to Jacobsen et al. [28] **,** there is a high rate (51%) of histological discordance between RPLND and liver lesion specimens, with 73% of liver lesions containing only necrosis.

The overall complication rate in this study (40 patients, 24.7%) is similar to the reports in the literature (12–32% of patients) [11,29,30,31,32]. Most of the studied patients experienced Grade I or II Clavien-Dindo complications, with only one Grade IIIb complication recorded. No Grade IV or V complications were recorded. The complication rate has been proven to correlate with the setting of RPLND (primary vs. pc-RPLND), with a higher rate of intraoperative and postoperative complications in the pc-RPLND series. Moreover, the extent of RPLND is another key factor determining postoperative complications of these patients. Recently, a large multicentre study in the Scandinavian population compared intra- and postoperative complications in patients who underwent unilateral or bilateral pc-RPLND [33]. Bilateral pc-RPLND was associated with a higher frequency of postoperative complications (45% vs. 25% in unilateral), while 8.3% of the patients had ≥ Grade 3b events (vs 2.2% in unilateral procedures). A recent systematic review comparing outcomes of different pc-RPLND techniques, confirms the higher incidence of major complications (Grade III-V) in patients undergoing full bilateral RPLND (18%) compared to complications associated with unilateral template RPLND (8%) [34]. This study shows that patients who underwent full bilateral RPLND had three times as many complications as those who underwent a template RPLND (*p* = 0.026, OR = 3.37 with 96% CI: 1.102–10.101).

Histology of the excised masses after pc-RPLND is a key determinant of prognosis in patients with viable GCT or teratoma. However, considering its high morbidity, pc-RPLND offers no benefit in patients with residual fibrosis or necrosis. In our centre, the rate of necrosis/fibrosis of the residual mass was 39.5%, which is a bit lower than the reported literature rates (47–67%) [15,35,36], while patients with teratoma represented 51.9% of cases, slightly higher than the reported rate of 30–40% [7] This may be attributed to the referral pattern of the centre and the fact that the majority of TGC patients receive aggressive chemotherapy regimens from their attending oncologists, resulting in the referral of those who have an absolute indication for pc-RPLND.

The study’s primary limitations, such as its retrospective, non-randomized, and unblinded design, must be emphasized. Conducted at a single centre, it predominantly reflects local experiences, with findings applicable only to open RPLND procedures. Additionally, the potential confounding effect of increasing surgeon and team experience should be considered, as surgical outcomes typically improve with greater case volume. However, despite these limitations, it is important to acknowledge that the major strength of this study is that comprises consecutive patients operated by the same surgeon and managed perioperatively by the same team over a 15-year period.

## 5. Conclusions

This real-world data study describes the surgical outcomes of pc-RPLND in a tertiary referral centre. It reflects current practice, which is consistently harmonized with national and international guidelines. Centralization of these procedures in high-volume centres is key to maximise safety and ensure the best possible oncological outcomes.

## Figures and Tables

**Table 1:** Patients’ demographics and baseline characteristics prior to RPLND.

Variable	Number of Patients (%)	Median (Q1–Q3)
Age at RPLND (years)	165 (100%)	30.5 (24.75–38.25)
BMI prior to RPLND (kg/m^2^)	165 (100%)	25 (23–28)
Ethnicity	Caucasian 100%	
Symptoms at diagnosis		
Painless testicular mass	136 (82.4%)	
Painful testicular mass	16 (9.6%)	
Back pain	11 (6.7%)	
Abdominal pain	2 (1.3%)	
Side of primary tumour		
Right	59 (35.7%)	
Left	106 (64.3%)	
Pathology of primary testicular tumour		
Mixed NSGCT	127 (76.9%)	
Pure Embryonal	13 (7.9%)	
Seminoma	11 (6.7%)	
Yolk sac carcinoma	7 (4.3%)	
Pure Choriocarcinoma	2 (1.2%)	
Teratoma	5 (3%)	
Prognostic Group		
IIA	16 (9.7%)	
IIB	31 (18.8%)	
IIC	49 (29.7%)	
III	69 (41.8%)	
Maximum transverse diameter of retroperitoneal masses prior at diagnosis (mm)	165 (100%)	51 mm (26–84.5)
Maximum transverse diameter of retroperitoneal masses prior to RPLND (mm)	165 (100%)	50 mm (26.75–81.25)
Indications of RPLND		
Normalized post-chemotherapy tumour markers	154 (93.3%)	
Plateaued	7 (4.2%)	
Salvage	4 (2.5%)	
Chemotherapy regimen prior to pc-RPLND		
BEP	125(75.8%)	
VIP	2 (1.2%)	
EP	7 (4.2%)	
BEP + VIP	4 (2.4%)	
BEP + TIP	26 (15.8%)	
VIP + TIP	1 (0.6%)	

RPLND: Retroperitoneal Lymph Node Dissection; NSGCT: Non-Seminomatous Germ-cell Tumour; AFP: alpha Fetoprotein. BEP: Bleomycin Etoposide Cisplatin, TIP: Paclitaxel ifosfamide cisplatin, EP: Etoposide cisplatin, VIP: cisplatin etoposide ifosfamide.

**Table 2:** Intraoperative parameters.

Variable	Number of Patients (%) (Patients in the Analysis n = 162)	Median (Q1–Q3)
Type of RPLND		
Full Bilateral	125 (77%)	
Modified template	37 (23%)	
Duration of the procedure		295 min (240–360 min)/ 4.9 h (4–6 h)
Estimated Blood Loss		300 mL (120–740 mL)
ClassIntra v1.0 classification of intraoperative adverse events		
Grade I	20 (12.4%)	
Grade II	8 (5%)	
Grade III	0	
Grade IV	0	
Grade V	1 (0.6%)	
Adjunctive procedures	38/162 (23.5%)	
Nephrectomy	18 (11.1%)	
Ureteric stent	3 (1.9%)	
Rupture of Right Internal Iliac Vein	1 (0.6%)	
Rupture of IVC	1 (0.6%)	
Ligation of IVC	4 (2.5%)	
Total IVC and Aorta replacement	1 (0.6%)	
Partial Vertebrectomy	1 (0.6%)	
Trans ureteroureterostomy	3 (1.9%)	
Partial Hepatectomy	3 (1.9%)	
Ureter replacment with ileus	1 (0.6%)	
Renal autotransplantation	1 (0.6%)	
Intraoperative ablation of Liver metastasis	1 (0.6%)	

RPLND: Retroperitoneal Lymph Node Dissection; IVC: Inferior Vena Cava.

**Table 3:** Association between type of RPLND and presence of complications.

Type of Surgery	Complications (N)		*p* Value
	Yes	No	
Bilateral RPLND	36	89	0.026
Modified template	4	33	

**Table 4:** Postoperative parameters and histopathological findings.

Variable	Number of Patients (%)	Median (Q1–Q3)
Postoperative complications according to Clavien-Dindo classification	40 (24.7%)	
Grade I		
Lymphatic leakage	28 (17.3%)	
Fever	3 (1.9%)	
Grade II		
Infection (Chest or wound)	4 (2.5%)	
Paralytic ileus	2 (1.2%)	
Pulmonary embolism/Deep Vein Thrombosis	2 (1.2%)	
Grade IIIb		
Wound Dehiscence	1 (0.6%)	
Length of hospital stay		8 days (7–10 days)
Histology of resected lymph nodes		
Seminoma	1 (0.6%)	
Pure Embryonal	2 (1.2%)	
Yolk sac carcinoma	2 (1.2%)	
Teratoma	84 (51.9%)	
Mixed NSCT	7 (4.4%)	
Fibrosis	64 (39.5%)	
Other (Rhabdomyosarcoma, nephroblastoma, adenocarcinoma)	2 (1.2%)	

## Data Availability

Data are contained within the article.
